# Supplementary material for: Repeated Infusions of Bone-Marrow-Derived Mesenchymal Stem Cells over 8 Weeks for Steroid-Refractory Chronic Graft-versus-Host Disease: A Prospective, Phase I/II Clinical Study
Source: Int J Mol Sci. 2024 Jun 19;25(12):6731. doi: 10.3390/ijms25126731 (PMC11204151; doi:10.3390/ijms25126731)
Supplement: Supplementary file 1 [file ijms-25-06731-s001.zip › ijms-3030388-supplementary.pdf]

Supplementary Table S1. Adverse events

Patient (UPN#)	Comments
1	No MSC related adverse events.
2	Fever (38.0°C) following first MSC infusion
3	Fever (38.0°C) following first MSC infusion
4	Grade 2 upper respiratory infection one month following last MSC infusion
5	No MSC related adverse events.
6	No MSC related adverse events.
7	No MSC related adverse events.
8	Grade 2 diarrahea during second MSC infusion
9	Fever (37.6°C) following second MSC infusion
10	No MSC related adverse events.

Supplementary Table S2. Lab Parameters

	0w			8w			18w			54w		
	**Responder**	**Non-responder**	**P value**	**Responder**	**Non-responder**	**P value**	**Responder**	**Non-responder**	**P value**	**Responder**	**Non-responder**	**P value**
**WBC**	8.82 (5.22-14.7)	8.89 (5.67-10.4)	0.972	9.20 (4.88-12.16)	8.97 (5.08-11.22)	0.901	9.97 (8.24-12.6)	8.61 (5.91-11.84)	0.405	8.13 (5.67-10.08)	9.10 (8.37-9.84)	0.472
**Lymphocyte, %**	30.46 (17.9-46.3)	38.6 (32.3-50.8)	0.254	33.1 (28.1-45.4)	29.67 (21.1-34.6)	0.482	28.64 (12.6-46.7)	32.57 (9.7-53)	0.725	36.72 (19.1-58.6)	37.9 (32.4-43.4)	0.924
**ALC**	3.12 (1.86-5.06)	2.85 (1.88-3.92)	0.737	3.19 (1.64-5.23)	2.57 (1.75-3.43)	0.428	3.04 (1.03-5.88)	2.52 (1.04-3.82)	0.651	2.93 (1.95-5.25)	3.41 (3.18-3.63)	0.655
**ANC**	5.16 (1.87-9.7)	5.3 (2.93-8.3)	0.942	5.27 (2.31-6.56)	5.54 (2.61-8.03)	0.847	6.61 (5.41-8.67)	5.08 (1.64-9.13)	0.366	4.51 (2.4-7.23)	4.62 (4.03-5.22)	0.949
**RBC**	4.32 (3.66-5.1)	3.82 (3.31-4.6)	0.220	4.37 (3.82-4.77)	3.95 (3.5-4.61)	0.162	4.46 (3.97-4.92)	4.19 (4.14-4.25)	0.138	4.48 (3.82-4.93)	3.87 (3.4-4.34)	0.178
**Hemoglobin**	14.17 (11.9-15.3)	12.55 (10.6-14.6)	0.120	14.15 (12.8-15.4)	12.77 (11.6-14.3)	0.093	14.23 (13.4-15.3)	13.47 (12.8-14.4)	0.182	14.33 (12.88-15.2)	12.35 (11.1-13.6)	0.077
**Hematocrit**	42.2	37.92 (32.5-44)	0.177	42.75	39.15	0.125	42.52	40 (38.3-42)	0.167	42.58	36.9	0.059

	(34.5-46.2)			(39.4-46.3)	(35.9-4.6)		(39.2-46.5)			(39.5-45.3)	(33.1-40.7)	
**Platelet**	268.5 (179-429)	243 (150-360)	0.673	318.67 (226-490)	248 (130-365)	0.296	286.83 (170-419)	219.5 (123-322)	0.248	284.33 (139-447)	366 (214-518)	0.462
**Glucose**	105.17 (88-167)	162 (99-242)	0.082	117.83 (89-162)	115.75 (21-240)	0.959	143.83 (85-281)	141.5 (106-173)	0.956	103.17 (85-147)	145 (101-189)	0.166
**BUN**	12.18 (7.9-21.4)	22.37 (14.5-26.1)	0.014	11.85 (6.4-23.4)	19.5 (11.5-23.2)	0.076	12.6 (7.2-26.4)	22.45 (13.9-25.2)	0.102	13.88 (8.6-23.9)	15.35 (8.3-22.4)	0.791
**AST**	40.83 (22-78)	37.25 (18-74)	0.806	35.17 (20-64)	35 (20-42)	0.988	31.83 (21-39)	32.25 (13.9-35.6)	0.960	30.67 (20-44)	44.5 (19-70)	0.351
**ALT**	57.83 (12-184)	38.75 (17-70)	0.584	53 (23-164)	34.25 (22-50)	0.528	37.5 (19-59)	47.5 (16-84)	0.529	32.83 (15-69)	35 (18-52)	0.902
**Total Bilirubin**	0.59 (0.36-0.77)	0.59 (0.45-0.96)	0.919	0.57 (0.46-0.66)	0.58 (0.41-0.87)	0.865	0.50 (0.37-0.6)	0.68 (0.42-1.04)	0.153	0.47 (0.33-0.74)	0.75 (0.48-1.02)	0.145
**Total Protein**	6.4 (5.1-7.5)	6.17 (5.3-7)	0.681	6.88 (6.3-7.5)	6.275 (5.9-7.1)	0.114	6.98 (6.3-7.9)	6.475 (5.8-7.1)	0.212	7 (5.9-7.6)	6.55 (6.1-7)	0.408
**LDH**	486.5 (350-590)	424.5 (373-523)	0.342	483.5 (244-649)	445 (328-676)	0.693	461.83 (290-594)	457.75 (309-732)	0.965	390.83 (87-610)	419 (383-455)	0.839
**CRP**	1.32 (0.02-4.52)	0.42 (0.06-1.16)	0.447	0.45 (0.02-1.91)	0.59 (0.04-.1.4)	0.779	0.085 (0.06-0.11)	0.98 (0.52-1.45)	0.193	0.097 (0.01-0.28)	1.22 (0.04-2.4)	0.198

**Albumin**	3.86 (2.8-4.4)	3.65 (3.4-4.1)	0.569	4.36 (3.9-4.9)	3.7 (3.2-4.2)	0.044	4.26 (3.9-4.8)	3.77 (3.2-4.3)	0.147	4.26 (4.1-4.6)	3.8 (3.2-4.4)	0.250

Abbreviations: ALC, absolute leukocyte count; ANC, absolute neutrophil count; AST, aspartate aminotransferase; ALT, alanine aminotransferase; BUN, blood urea nitrogen; CRP, c-reactive protein; LDH, lactate dehydrogenase; RBC, red blood cell ; W, weeks; WBC, white blood cell

Values indicate mean(range). Responders included 6 patients (UPN-1, 2, 3, 4, 9, 10) and non-responders included 4 patients (UPN-5, 6, 7, 8).

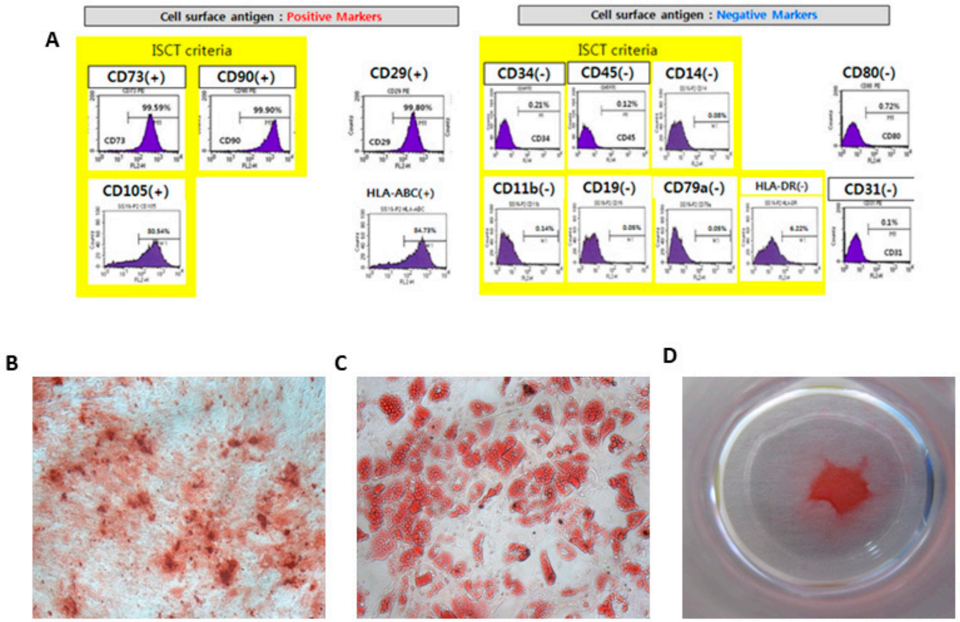

Supplementary Figure S1. Characteristics of infused bone-marrow derived mesenchymal stem cells. Infused bone-marrow derived mesenchymal stem cells (BM-MSCS) were analyzed for (A) cell surface antigens based on the international society of cellular therapy (ISCT) minimal criteria for defining MSCs. BM-MSCs showed trilineage differentiation to (B) osteoblasts (left), (C) adipocytes (center), and (D) chondrocytes (right) *in vitro*.

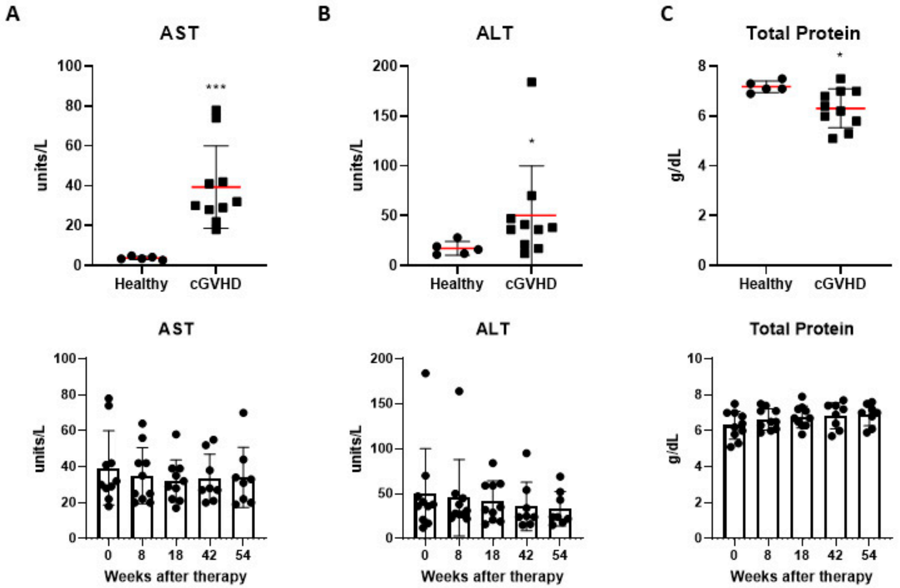

Supplementary Figure S2. Lab parameters considered in chronic graft-versus-host disease patients. Blood chemistry tests were performed throughout the study. Few components, including ALT (A), AST (B) and total protein (C) were significantly different in cGVHD patients (n=10) compared to healthy controls (n=5). P-values for comparing cGVHD and healthy controls with t test. *p, 0.05, ***, p<0.0005. The changes in these levels were followed during follow up. ALT, alanine aminotransferase; AST, aspartate aminotransferase; cGVHD, chronic graft-versus-host disease.

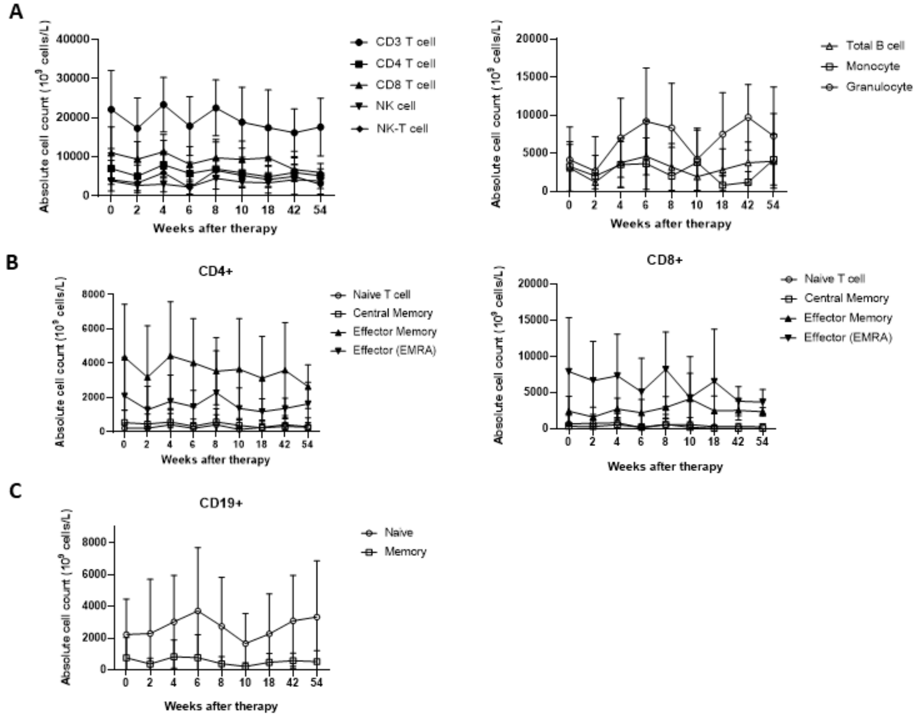

Supplementary Figure S3. Serial monitoring of immune cell subsets during follow-up. Flow cytometric analyses of (A) different lymphocyte subsets, (B) memory T cell subsets, and (C) B cell subsets over 54 weeks of follow-up period after mesenchymal stem cell (MSC) infusion. 10 patients were evaluated on week 0, week 8, and week 18; and 8 patients were evaluated on week 42 and week 54 following the first MSC infusion. EMRA, terminally differentiated effector memory; NK, natural killer cells; NKT, natural killer like T cells.
